# μ-Coriolis Mass Flow Sensor with Resistive Readout

**DOI:** 10.3390/mi11020184

**Published:** 2020-02-11

**Authors:** Thomas Schut, Remco Wiegerink, Joost Lötters

**Affiliations:** 1 MESA+ Institute for Nanotechnology, University of Twente, P.O. Box 217, 7500 AE Enschede, The Netherlands; r.j.wiegerink@utwente.nl (R.W.); j.c.lotters@utwente.nl (J.L.); 2 Bronkhorst High-Tech BV, Nijverheidsstraat 1A, 7261 AK Ruurlo, The Netherlands

**Keywords:** coriolis, flow sensor, flow, mass flow, strain gauge, resistive, resonator, channel

## Abstract

This paper presents a μ-Coriolis mass flow sensor with resistive readout. Instead of measuring a net displacement such as in a capacitive readout, a resistive readout detects the deformation of the suspended micro-fluidic channel. It allows for actuation at much higher amplitudes than for a capacitive readout, resulting in correspondingly larger Coriolis forces in response to fluid flow. A resistive readout can be operated in two actuation vibrational modes. A capacitive readout can only be operated in one of these two modes, which is more sensitive to external disturbances. Three types of devices have been realized. We present measurement results for all three devices. One device clearly outperforms the other two, with a flow sensitivity of 2.22 °/(g·h^−1^) and a zero-flow stability of 0.02 g·h^−1^ over 30 min. Optimization of the metal strain gauges and/or implementation of poly-Silicon strain gauges could further improve performance.

## 1. Introduction

A μ-Coriolis mass flow sensor measures true mass flow, independent of fluid properties. A free-hanging micro-channel is brought into resonance in a certain vibrational mode. A flow through the channel causes Coriolis forces, inducing a second mode of vibration. The ratio of the magnitudes of the two modes is a measure for the flow. These vibrations can be measured in multiple ways, e.g., optical, capacitive or resistive (strain gauges). An optical detection method requires either an external light source [1] or on-chip waveguide structures [2,3] usually used for in-plane vibrating devices and complicated to integrate into the fabrication process of a μ-Coriolis mass flow sensor. A capacitive readout has been shown to be a good method by Haneveld et al. [4], Sparks et al. [5], and by Groenesteijn et al. [6] with the most sensitive μ-Coriolis flow sensor to date. However, a capacitive readout has its limitations, such as limited actuation amplitude, pressure dependency, and influence of relative permittivity of the fluid. These limitations are explained in more detail in Section 2.2. A resistive readout based on strain gauges on the micro-channel does not suffer from the aforementioned limitations. This paper focuses on the implementation of strain gauges with a μ-Coriolis flow sensor as an alternative to a capacitive readout.

## 2. Materials and Methods

### 2.1. Operating Principle

**Figure 1:**
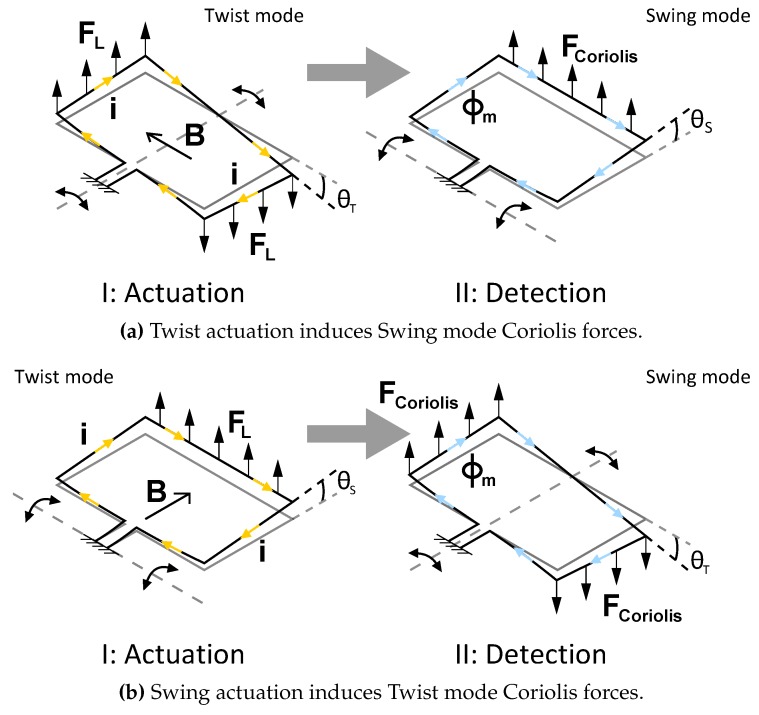
Operation principle of a micro-Coriolis mass flow sensor. I: The channel loop is brought into resonance with actuation angle $\theta_{T}$ in (**a**), $\theta_{S}$ in (**b**) through Lorentz force $F_{L}$, resulting from a magnetic field B and an AC current *i*. II: A mass flow $\phi_{m}$ through the channel induces vibration in the detection mode through Coriolis force $F_{\mathrm{Coriolis}}$, resulting in a sensing angle $\theta_{S}$ in (**a**), $\theta_{T}$ in (**b**). The ratio between the two vibration mode amplitudes is a measure for the flow.

Figure 1 shows the operation principle of a μ-Coriolis mass flow sensor. The sensor consists of a rectangular channel loop. The channel is fixed at the in-/outlet channel sections, as depicted in the figure. The channel loop is brought into resonance by Lorentz forces. These Lorentz forces are generated by a magnetic field B and an alternating current *i* flowing through a metal track on top of the micro-channel. This actuates the sensor in the *Twist*/actuation mode with actuation angle $\theta_{a}$. When a fluid flows through the micro-channel with a mass flow rate $\phi_{m}$, Coriolis forces are induced in the channel section as indicated in Figure 1. This causes additional vibration in the *Swing*/detection mode with an angle $\theta_{d}$ and displacement $\Delta z_{d}$. The ratio $\Delta {\hat{z}}_{d}/{\hat{\theta }}_{a}$ is a measure for the flow.

### 2.2. Capacitive Readout

**Figure 2:**
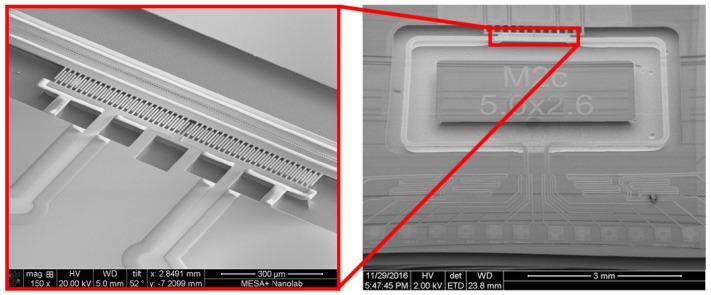
SEM image of a μ-Coriolis mass flow sensor with capacitive readout. The capacitive readout consists of two comb capacitor structures used for measuring displacement of the micro-channel.

**Figure 3:**
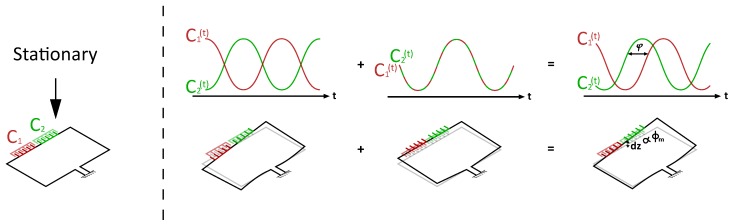
Operation principle of the capacitive readout. When actuated in Twist mode, the capacitance signals over time are 180${}^{\circ }$ out of phase. Swing mode vibration induced by a mass flow through the channel causes common mode signals. When actuating with flow, the capacitance signals will have a phase difference φ dependent on the mass flow rate $\phi_{m}$.

μ-Coriolis mass flow sensors utilizing surface channels [7] with capacitive readout have been presented previously [4,6]. Figure 2 shows an SEM image of such a μ-Coriolis mass flow sensor, indicating the capacitive readout structures. They are centered around the rotational axis of the Twist vibrational mode. Figure 3 shows the operation principle of the capacitive readout. For the capacitive readout, the sensor is typically actuated in the Twist mode. When actuated, the capacitance signals over time are 180${}^{\circ }$ out of phase. When a fluid flows through the channel, Swing mode vibrations are induced causing common mode capacitance signals. During operation with flow, the sensor is actuated and a mass flow flows through the channel. This gives a combination of Twist and Swing mode vibrations. The capacitance signals will have a phase difference φ dependent on the mass flow rate $\phi_{m}$.

**Figure 4:**
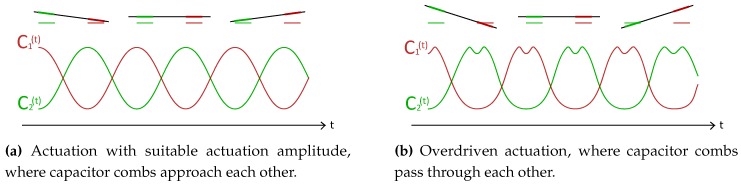
Schematic representation of the actuation limit for a capacitive readout. The channel section with comb structures floats slightly above the fixed comb structures when stationary due to residual stress in the channel. Large actuation amplitude (**b**) gives nonlinearity in the capacitance signals, limiting the signal-to-noise ratio.

As aforementioned, a capacitive readout has certain limitations. For one, the actuation amplitude is limited by inherent nonlinearity [8]. This limits the signal-to-noise ratio of the device. When stationary, the channel section to which the capacitor combs are attached floats slightly above the combs fixed to the substrate. This is due to residual stress in the silicon nitride channel developed during fabrication. The distance is in the order of a few μm. This means that when the actuation amplitude is high enough that the channel moves more than this distance, the capacitor combs will pass through each other. Figure 4 shows how this causes nonlinearity in capacitance.

An additional effect of nonlinearity of the capacitive readout is that Swing mode actuation is not feasible. For Twist mode actuation, the capacitive readout structures are put as close to the Twist mode rotational axis as possible to maximize sensitivity to Swing mode Coriolis vibrations. Thus, for Swing mode actuation, one would like to maximize sensitivity for Twist mode Coriolis vibrations. However, the largest displacements in both modes occur in the top corners of the channel loop (see Figure 3). Putting the readout structures here would limit the actuation amplitude even more, since relatively large displacements due to actuation occur here. Therefore, Twist mode actuation is typically used for these devices. However, external vibrations can more easily induce vibrations in the Swing mode. When the Swing mode is used as Coriolis mode, small disturbances can have a significant effect on the output signal, since Coriolis displacements are relatively small (in the order of 10–100 nm). Because of this fact, it would be preferable to actuate the sensor in the Swing and detect Twist mode Coriolis forces.

Another effect is that pressure inside the channel causes the channel to bend upwards, due to its non-circular cross-section. This results in a change in distance between the capacitor combs, which shifts the point of nonlinearity (see Figure 4). This phenomenon is explained by Alveringh et al. [8]. Furthermore, relative permittivity of the fluid flowing through the channel influences the measured capacitance.

### 2.3. Resistive Readout

The resistance of a material is defined as:

(1) $$R=\rho \frac{l}{A},$$

where ρ is the material’s resistivity, *l* is the length of the material, and *A* is its cross-sectional area. For a metal strain gauge, ρ remains constant with deformation. The change in the ratio l/A solely determines the change in resistance of the strain gauge, as opposed to piezo-resistive materials where ρ changes with deformation of the material. The resistive readout presented in this paper is based on metal strain gauges, since their implementation requires no changes to the fabrication process of the μ-Coriolis mass flow sensor. After a first proof of principle, poly-silicon strain gauges could be used to significantly increase sensitivity to strain (explained in more detail in Section 4).

**Figure 5:**
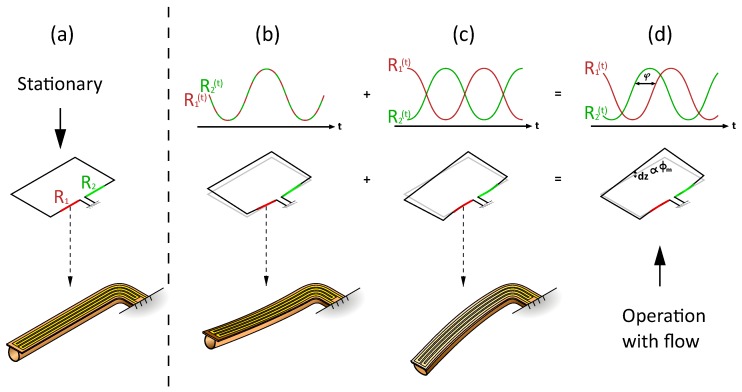
Principle of operation of the resistive readout. (**a**) configuration of the strain gauges in rest. (**b**) In the *Swing* mode, both strain gauges will be compressed. This results in equal resistance change. (**c**) In the *Twist* mode, one strain gauge is elongated, the other will be compressed. This results in opposite change in resistance. (**d**) Combination of the two modes gives two resistance signals with a phase shift dependent on the mass flow rate.

Figure 5 shows the operating principle of the resistive readout. In this example, the sensor is actuated in the *Swing* mode. Figure 5a shows the configuration of the strain gauges when the sensor is not being actuated. When the sensor is actuated (Figure 5b), both strain gauges are elongated at the same time and compressed at the same time. The resistances over time of the two strain gauges are thus in phase for the two strain gauges. When there is a flow through the channel, Coriolis forces induce vibration in the *Twist* mode; see Figure 5c. This vibration is 90 degrees out of phase relative to the actuation [4]. One strain gauge is elongated while the other is compressed. The resistances over time of the strain gauges are thus in opposite phase and ±90 degrees out of phase with the resistance change due to actuation. When combining actuation and Coriolis vibrations, the resistances of the strain gauges will have a phase difference which depends on the mass flow rate through the channel. This method of readout is advantageous, since it does not depend on the amplitudes of the resistive signals but only the phase. Temperature dependence in the amplitudes of the signal could be more significant for a capacitive readout. However, since the phase of the signals is measured, temperature dependence of the resistive readout should be comparable to that of a capacitive readout.

### 2.4. Design

**Figure 6:**
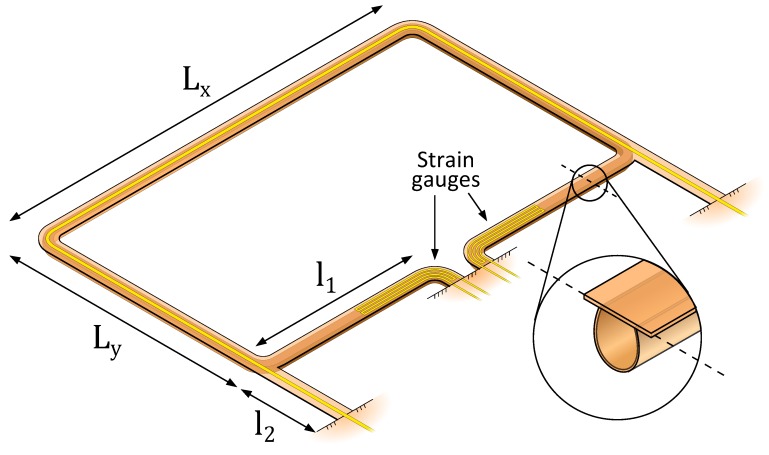
Design A/B: Two strain gauges are positioned on the in-/outlet channel sections. For design A, the strain gauge is designed to measure torsion of the channel section with length $l_{1}$ around its axis. For design B, the strain gauge is designed to measure bending of the same channel section along its axis. Two membranes with length $l_{2}$ connect the channel loop to the substrate to allow for the metal actuation track.

**Figure 7:**
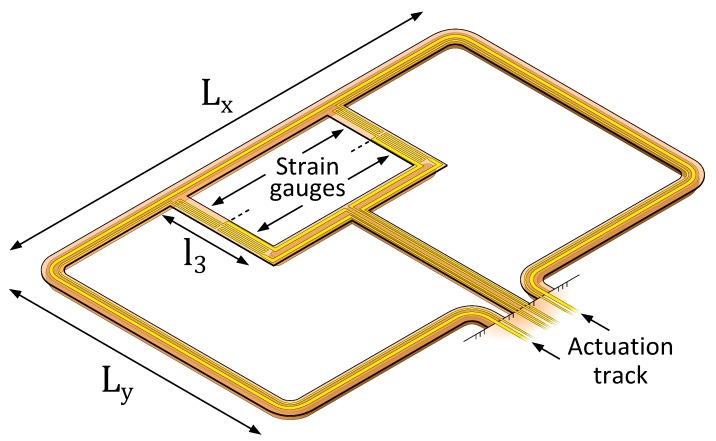
Design C: A T-junction channel structure is connected through two membranes to the main channel loop. Four strain gauges reside on the membranes with length $l_{3}$. Two experience elongate, while the other two experience compression.

Three designs of μ-Coriolis mass flow sensors with resistive readout were made, based on two channel loop structures. The sensors are designed to operate up to a mass flow rate corresponding to a pressure drop of 1 bar over the sensor for water flow. This is approximately 1.8 g·h^−1^. Figure 6 and Figure 7 show the two channel structures. Both designs are based on a channel loop of $L_{x}\times L_{y}=5\times 3$ mm, which are typical dimensions of a μ-Coriolis mass flow sensor. Designs A and B share the same channel loop structure (Figure 6). The strain gauges in both designs reside on the channel sections with length $l_{1}$. Additional membranes with length $l_{2}$ connect the channel loop to the substrate to allow for the actuation track. For design A, the strain gauges are patterned at a 45${}^{\circ }$ angle to measure torsion of the channel section with length $l_{1}$. This makes the design relatively more sensitive to Swing mode vibrations. Thus, this design is designed for Twist mode actuation (Swing mode Coriolis forces). For Design B, the strain gauges are patterned parallel to the channel section with length $l_{1}$ to measure bending along its axis. This makes the device relatively more sensitive to Twist mode vibration. Thus, it is designed for Swing mode actuation (Twist mode Coriolis forces). Microscopic photographs of the fabricated devices in Section 2.5 will show the difference between the two designs more clearly. Design C is based on a T-junction channel structure connected through two membranes to the main channel loop (Figure 7). This way, a location where stress is induced is created by design. Four strain gauges reside on the two membranes with length $l_{3}$. Two of them will elongate, while the other two are compressed. This design can be used for both modes of actuation.

**Figure 8:**
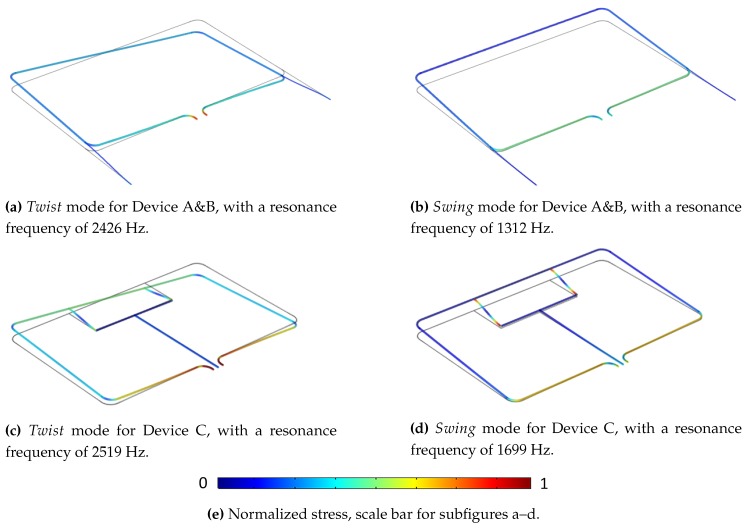
Vibrational modes for the different device types, simulated in COMSOL Multiphysics. (**a**,**b**) Show that stress is the highest at the channel sections with length $l_{1}$. This is why designs A and B have strain gauges in these sections, optimized for Swing/Twist mode actuation respectively; (**c**,**d**) show the stress in the connecting membranes. Based on these simulations, device C was designed with four strain gauges as shown in Figure 7.

The vibrational modes for the different devices were simulated in COMSOL Multiphysics and are depicted in Figure 8. The Twist/Swing mode resonance frequencies are 2426/1312 Hz respectively for device A&B. The stress is highest in the channel sections with length $l_{1}$. Therefore, the two designs were made as aforementioned, optimized for Twist/Swing mode actuation respectively. For design C, the Twist/Swing mode resonance frequencies are 2519/1699 Hz respectively (Figure 8c–d). The stress is highest where the two membranes with length $l_{3}$ are attached to the main channel loop and the T-junction channel structure. The sensitivity to either mode of actuation could be tuned by increasing/decreasing the distance between the two membranes, increasing/decreasing the sensitivity to Twist mode vibration.

### 2.5. Fabrication

**Figure 9:**
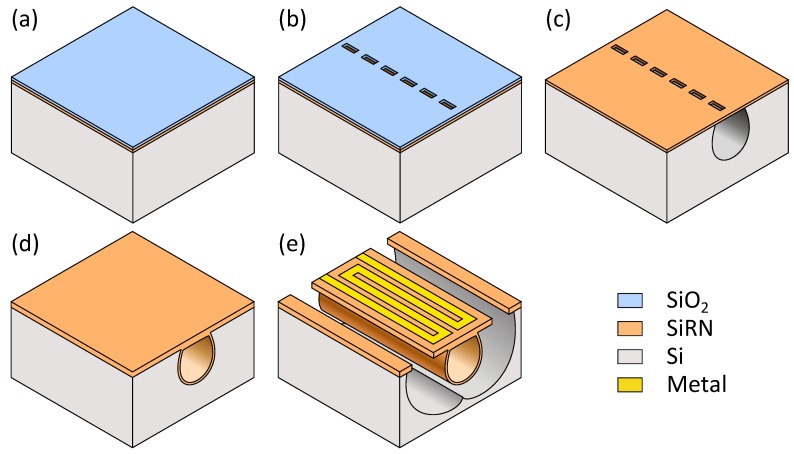
Fabrication process for *Surface Channel Technology*. (**a**) silicon wafer with 500 nm SiRN and 500 nm SiO_2_; (**b**) patterning of slit openings; (**c**) removal of SiO_2_ hard mask and forming of the surface channel; (**d**) closing of the channel; (**e**) patterning of metal and release of the channel.

Integration of resistive strain gauges on top of the sensor tube is rather straightforward. The fabrication process of the sensor is based on the *Surface Channel Technology* (SCT) proposed in [7]. This is the same technology as used for μ-Coriolis mass flow sensors with capacitive readout [4,6]. A simplified schematic representation of the fabrication process is displayed in Figure 9. First, a layer of low stress silicon-rich silicon nitride (SiRN) (Thickness: 500 nm) is deposited on a Silicon wafer by LPCVD, see Figure 9a. On top of this, a layer of silicon di-oxide (SiO_2_) is deposited (Thickness: 500 nm), serving as a hard mask. Slits of 5 × 2 μm are etched through both layers by plasma etching, see Figure 9b. Then, the layer of SiO_2_ is removed and a channel is formed by semi-isotropically etching silicon through the slits, see Figure 9c. This is done by plasma etching as well. The channel is closed by conformally depositing another layer of low stress SiRN, see Figure 9d. Metal strain gauges are patterned on top of the channel (Figure 9e). Following this, openings are etched through the nitride and finally the channel is released by isotropic etching of silicon by an SF_6_ plasma.

**Figure 10:**
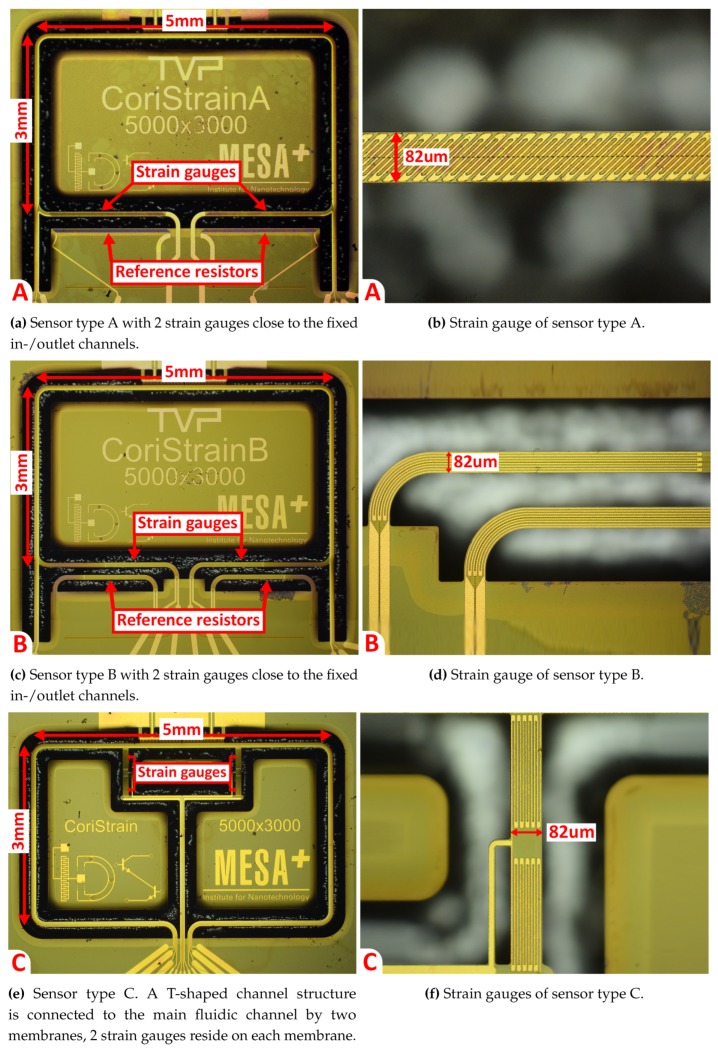
Microscope images of the three sensor types. Strain gauges and reference resistors are indicated in the subfigures. The metal tracks of the gauges are 4μm wide. The mask design of the strain gauges and reference resistors are identical.

Figure 10 shows microscope images of three sensor types that were designed and fabricated. The position of the strain gauges and reference resistors are indicated in the figure. The resistance value ratios between the devices are 1:0.73:0.37 (A:B:C) based on designed geometry. For all three devices, the sensor can in principle be actuated in either the Twist or Swing mode. However, sensitivity to flow differs throughout the devices and between actuation modes. As previously mentioned device A (Figure 10a,b) is designed for Twist mode actuation. Coriolis vibrations in the Swing mode are expected to induce torsion of the channel sections with length $l_{1}$, see Figure 8a,b. The strain gauges are patterned at a 45${}^{\circ }$ angle with respect to the channel, in order to measure this torsion. Device B (Figure 10c,d) is designed for Swing mode actuation. Coriolis vibrations in the Twist mode will cause bending of the channel sections as depicted in Figure 5 and Figure 8a,b. Finally, device C (Figure 10e,f) is designed to be used in either mode of actuation. Both Twist and Swing mode vibrations will cause elongation/compression of the strain gauges in a similar way, see Figure 8c,d. Therefore, the flow sensitivity of the device is expected to be similar for both modes of actuation. The sensitivity can be tuned by changing the distance between the two membranes on which the strain gauges reside. The larger this distance, the larger the sensitivity to Twist mode vibrations and thus relatively lower sensitivity to Swing mode vibrations.

### 2.6. Readout Electronics

**Figure 11:**
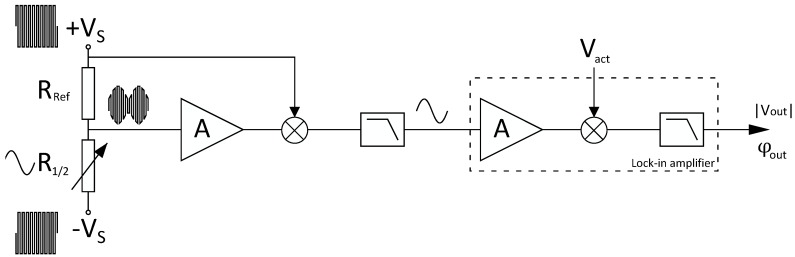
Schematic representation of the electronic readout circuit. The resistance of one strain gauge $R_{1/2}$ is converted to a voltage via a 1 MHz carrier signal and a reference resistor $R_{\mathrm{Ref}}$. In the case of device C (Figure 10e,f), $R_{\mathrm{Ref}}$ is another strain gauge with its resistance changing in the opposite direction with respect to $R_{1/2}$. The carrier signal is mixed out after which an SR860 lock-in amplifier determines the magnitude and phase of the signal (with actuation voltage $v_{\mathrm{act}}$ as a frequency reference).

The resistance of the strain gauges is read out using a circuit as depicted in Figure 11. $R_{1/2}$ represents either the left or the right strain gauge on the sensor. The resistance of one strain gauge $R_{1/2}$ is converted to a voltage via a high frequency (∼1MHz) carrier signal and an on-chip reference resistor $R_{\mathrm{Ref}}$. In the case of device C, $R_{\mathrm{Ref}}$ is another strain gauge with its resistance changing in the opposite direction with respect to $R_{1/2}$. The carrier signal is mixed out after which an SR860 lock-in amplifier determines the magnitude and phase of the signal. This is done by first amplifying the signal. Then, the signal is multiplied by the actuation voltage $v_{\mathrm{act}}$ and finally a low-pass filter filters out higher harmonics. Each strain gauge is read out with a circuit such as depicted in Figure 11. The phase difference between the two resulting signals is a measure for the mass flow rate.

### 2.7. Measurement Setup

**Figure 12:**
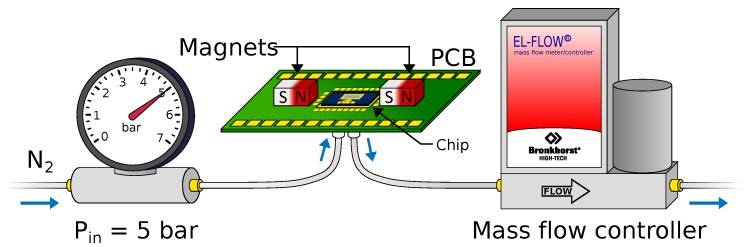
Fluidic measurement setup. Nitrogen gas is fed through the sensor chip at an input pressure $P_{\mathrm{in}}=5\;\mathrm{bar}$ (gauge pressure), while the mass flow is regulated using an external mass flow controller at the outlet. Figure adapted from [9].

Figure 12 shows the fluidic measurement setup. Nitrogen gas is fed through the sensor chip at an input pressure $P_{\mathrm{in}}$ of 5 bar (gauge pressure). The mass flow rate is controlled between 0–0.6 g·h^−1^ by a mass flow controller at the outlet of the sensor chip.

## 3. Results

The resistances of the strain gauges were measured and are ∼1100/800/460 Ω for design A/B/C, respectively. With ratios between the resistances of 1:0.73:0.42 (A:B:C), this corresponds to the theoretical resistance ratios presented in Section 2.5.

As previously mentioned, device A was designed for Twist mode actuation. However, measurements showed that the device was not sensitive to Swing mode Coriolis vibrations. The expectation was that Swing mode vibrations would cause significant torsion of the channel sections on which the strain gauges reside as depicted in Figure 8b. However, Laser Doppler Vibrometer measurements showed that the upwards motion of the channel in the Swing mode is mostly facilitated by bending of the channel section with length $l_{1}$ along its axis as opposed to torsion. This caused the strain gauges of device A to be sensitive to flow only when actuated in the Swing mode. For device B, the sensor was only sensitive to flow when actuated in the Swing mode, as expected. Device C was actuated in both modes and showed sensitivity to flow for both the Twist and Swing mode.

**Figure 13:**
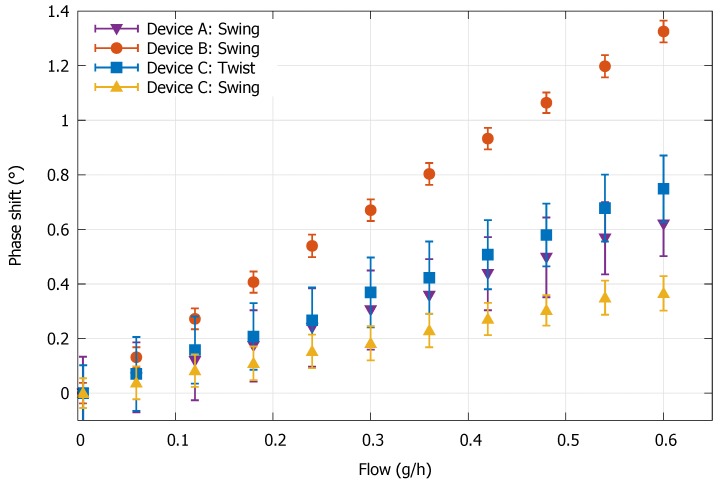
Output of the resistive readout in relation to the applied flow. Each shown measurement point is an average of the output signal over a period of 200 s at a stable flow. The error bars represent the standard deviation, which is determined over the same time period.

Flow measurements are executed with all three device types. The integration constant of the low-pass filter on the SR860 (Section 2.6) was set to 100 ms and its slope was set to 24 dB/decade. After sampling, the data it is averaged with a walking average over a time interval of 1 s. Figure 13 shows the resulting phase difference output of the resistive readout for the three device types.

**Figure 14:**
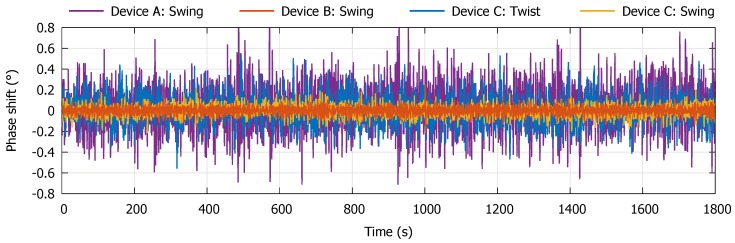
Phase difference output of the resistive readout with no flow over a period of 1800 s. The sensor’s channel is kept at a constant pressure of 5 bar (gauge pressure) while its outlet is closed off.

There is a linear relation between the output and mass flow rate for each device. Linearity for larger flows has not been investigated, as accuracy at small flows is of most interest. As can be seen from Figure 13, device type B shows the most stable output signal and also shows the highest sensitivity to flow. For each device, a zero-flow measurement has been executed to determine the devices’ zero stability over time. The outlet of the flow sensor was closed off and the channel of the sensor was kept at a constant pressure of 5 bar (gauge pressure). The results are displayed in Figure 14.

Device B shows the highest stability whereas the stability of device A is an order of magnitude lower. The latter was to be expected since device A was in principle designed to be actuated in the Twist mode and is thus not optimized for Swing mode actuation.

## 4. Discussion and Conclusions

All experimental parameters of the devices are displayed in Table 1. When comparing all devices, it is directly clear that device B shows the best overall performance. Its zero-flow stability is a factor 5 better than for device C, indicating stress during operation is much higher in the channel sections with length $l_{1}$ than the membranes with $l_{3}$. For device C, flow sensitivity for Twist mode actuation is approximately a factor 2 higher than for Swing mode actuation. However, the standard deviation and zero-flow stability of the phase output signal is around two times higher as well, compensating for the larger flow sensitivity (see Figure 14). This results in nearly equal standard deviation and zero-flow stability of the flow signal (in g·h^−1^). Since the zero-flow stability and standard deviation of the phase output signal is lower for Swing mode actuation, one could try to improve the sensitivity to Twist mode Coriolis forces to improve the performance of the device. This can be done by increasing the distance between the membranes on which the strain gauges reside, as previously mentioned.

The resistive readout shows great promise for improving the resolution and accuracy of the μ-Coriolis mass flow sensor. Sparreboom et al. [10] presented a μ-Coriolis based on a capacitive readout with a zero-flow stability of 2 mg·h^−1^. However, it is not clearly stated what sampling time is used. Since averaging the data can lead to lower standard deviation, the sampling time is important when comparing stability results. Since this is typically not stated, taking 2 mg·h^−1^ as a reference, the zero-flow stability of the resistive readout is a factor 10 lower. Optimization of the device can lead it to outperform the capacitive readout in the future. This can be done by optimizing the design of the sensor and the strain gauges. Another option is to use a piezo-resistive material to increase the sensitivity to strain significantly, e.g., doped mono-crystalline Silicon as proposed in [11]. Doped poly-silicon strain gauges on top of the channel of the sensor are also an option. The gauge factor of p-doped mono crystalline silicon ranges from −75 to +150 [12], whereas, for doped poly-silicon, the gauge factor ranges from −10 to +30 [12,13] depending on the doping concentrations. Although poly-silicon has a lower gauge factor than mono-crystalline silicon, it is more simple to implement and fabricate into a desired pattern on top of the microfluidic channel. Both options will be investigated for future designs as well as optimization of the current metal film strain gauges.

## Figures and Tables

**Table 1:** Experimental parameters of the three flow sensors determined from the (zero-)flow measurements. The zero-flow stability is defined as the standard deviation of the measured flow over a period of 1800 s (see Figure 14).

Device	Actuation	Resonance Frequency (*Model*/Measured) [Hz]	Flow Sensitivity [°/g·h^−1^]	Standard Deviation [g·h^−1^]	Zero-Flow Stability [g·h^−1^]
A	*Twist*	*2426* /2113	-	-	-
	*Swing*	*1312* /1184	1.08	0.12	0.19
B	*Twist*	*2426* /2156	-	-	-
	*Swing*	*1312* /1203	2.22	0.02	0.02
C	*Twist*	*2519* /2254	1.23	0.10	0.11
	*Swing*	*1699* /1624	0.63	0.10	0.09

## Abbreviations

The following abbreviations are used in this manuscript:

SEM	Scanning Electron Microscope
SCT	Surface Channel Technology
SiRN	Silicon-rich Silicon Nitride
LPCVD	Low-Pressure Chemical Vapor Deposition
